# Functional and physical interactions between P2Y receptors and ion channels

**DOI:** 10.1186/2050-6511-13-S1-A61

**Published:** 2012-09-17

**Authors:** Hend Gafar, Giri K Chandaka, Stefan Boehm

**Affiliations:** 1 Department of Neurophysiology and Neuropharmacology, Center for Physiology and Pharmacology, Medical University of Vienna, 1090 Vienna, Austria

## Background

Neuronal P2Y receptors, i.e. nucleotide-sensitive G protein-coupled receptors (GPCRs), are known to control various voltage-gated ion channels, in particular K_V_7 K^+^ and Ca_V_2.2 Ca^2+^ channels. The differential modulation of these ion channels via GPCRs was shown to rely on the presence or absence of scaffolding proteins such as AKAP79/150 and NHERF-2. Since scaffold proteins are believed to bring GPCRs and ion channels in close proximity to guarantee efficient G protein-mediated modulation, this project evaluates whether a tight contact between P2Y receptors and ion channels is a prerequisite for their functional interaction.

## Methods

P2Y_1_ or P2Y_12_ receptors with fluorescent tags (CFP or YFP) were expressed together with fluorescently labeled K_V_7.2/7.3 or Ca_V_2.2 channels in tsA 201 cells and the channel modulation by nucleotides was determined by measuring the according currents. To evaluate the behavior of the receptors and channels in the membrane, fluorescence recovery after photobleaching (FRAP) was determined by confocal laser microscopy.

## Results

Activation of P2Y_1_ but not of P2Y_12_ receptors by ADP inhibited the K^+^ currents in a concentration-dependent manner by up to 20.5 ± 1.9%. Conversely, activation of both, P2Y_1_ and P2Y_12_ receptors, reduced the Ca^2+^ currents by up to 60.1 ± 7.4% and 76.3 ± 4.2%, respectively. In initial FRAP experiments, the YFP-labeled receptors showed similar half-times of around 80 seconds. Upon coexpression of the P2Y_1_ receptor with the K_V_7 channel the half-time increased significantly (p < 0.009) to 116 seconds compared to single expression of receptor or channel only. In the case of P2Y_12_, coexpression with K_V_7 showed no significant change compared to P2Y_12_ or K_V_7 alone.

## Conclusions

These findings suggest that distinct ion channels are modulated by different P2Y receptors. K_V_7 currents are inhibited by P2Y_1_ whereas Ca_V_2.2 currents are reduced by both P2Y_1_ and P2Y_12_. Additionally, FRAP data show that the presence of K_V_7 slows down the movement of P2Y_1_ in the membrane, but not that of P2Y_12_. This suggests that there is a physical interaction between P2Y_1_ and K_V_7 which is not present between P2Y_12_ and K_V_7. The influence on movement of P2Y_1_ and P2Y_12_ receptors in the membrane by the presence Ca_V_2.2 remains to be elucidated.

